# Age-related change in skeletal muscle strength, size, and adiposity over 18 years in Caribbean men

**DOI:** 10.1007/s11357-025-02023-8

**Published:** 2025-12-10

**Authors:** Lauren S. Roe, Ryan K. Cvejkus, Victor W. Wheeler, Christopher L. Gordon, Tanya S. Kenkre, Joseph M. Zmuda, Iva Miljkovic

**Affiliations:** 1 Division of Geriatric Medicine, Department of Medicine, University of Pittsburgh School of Medicine, 130 N Bellefield Ave, Suite 300, Pittsburgh, PA 15213 USA; 2 Department of Epidemiology, University of Pittsburgh, Pittsburgh, PA USA; 3 Tobago Health Studies Office, Scarborough, Tobago Trinidad and Tobago; 4 Department of Radiology, McMaster University, Hamilton, ON Canada

**Keywords:** Myosteatosis, Intermuscular adipose tissue, Grip strength, Epidemiology, PQCT

## Abstract

Declines in muscle strength outpace declines in muscle mass with age, so factors like muscle adiposity (i.e., myosteatosis) may contribute to strength loss. We describe intermuscular adipose tissue (IMAT) and grip strength over 18 years among middle-aged and older African Caribbean men. We hypothesized that increases in IMAT precede the loss of muscle area and strength, and that increased IMAT is independently associated with strength decline. Men (*n* = 339, 51.2 ± 5.5 years) were followed for 18.2 ± 0.9 years. Peripheral quantitative computed tomography at the calf determined IMAT (cm^2^) and muscle area (cm^2^) at baseline, follow-up visit 1 (FV1; 6.2 ± 0.4 years after baseline), and FV2 (12.0 ± 0.8 years after FV1). Grip strength (kg) was measured by handheld dynamometry at all visits. Generalized estimating equations determined associations between changes in IMAT and muscle area with time-varying grip strength. Grip strength decreased (mean ± SD, baseline: 51.33 ± 8.79, FV1: 47.02 ± 8.67, FV2: 40.12 ± 8.57), IMAT increased (median [quartile 1, quartile 3], baseline: 1.36 [0.73, 2.53], FV1: 1.74 [0.92, 3.14], FV2: 3.49 [2.06, 5.49]), and muscle area increased then decreased (baseline: 79.88 ± 12.33, FV1: 81.12 ± 13.88, FV2: 78.35 ± 12.72; all *p* < 0.05). A 1-cm^2^ increase in IMAT from baseline to FV1 or FV2 was significantly associated with 0.18 kg (95% CI: −0.31, −0.06) decreased grip strength, independent of muscle area. The change in muscle area was not associated with grip strength declines (*β* = 0.03, 95% CI: −0.03, 0.10). Despite initial muscle area increases, grip strength continuously decreased, and IMAT increased. Age-related increases in IMAT may contribute to the disconnect between declines in muscle strength and size.

**Graphical Abstract:**

**Figure:**
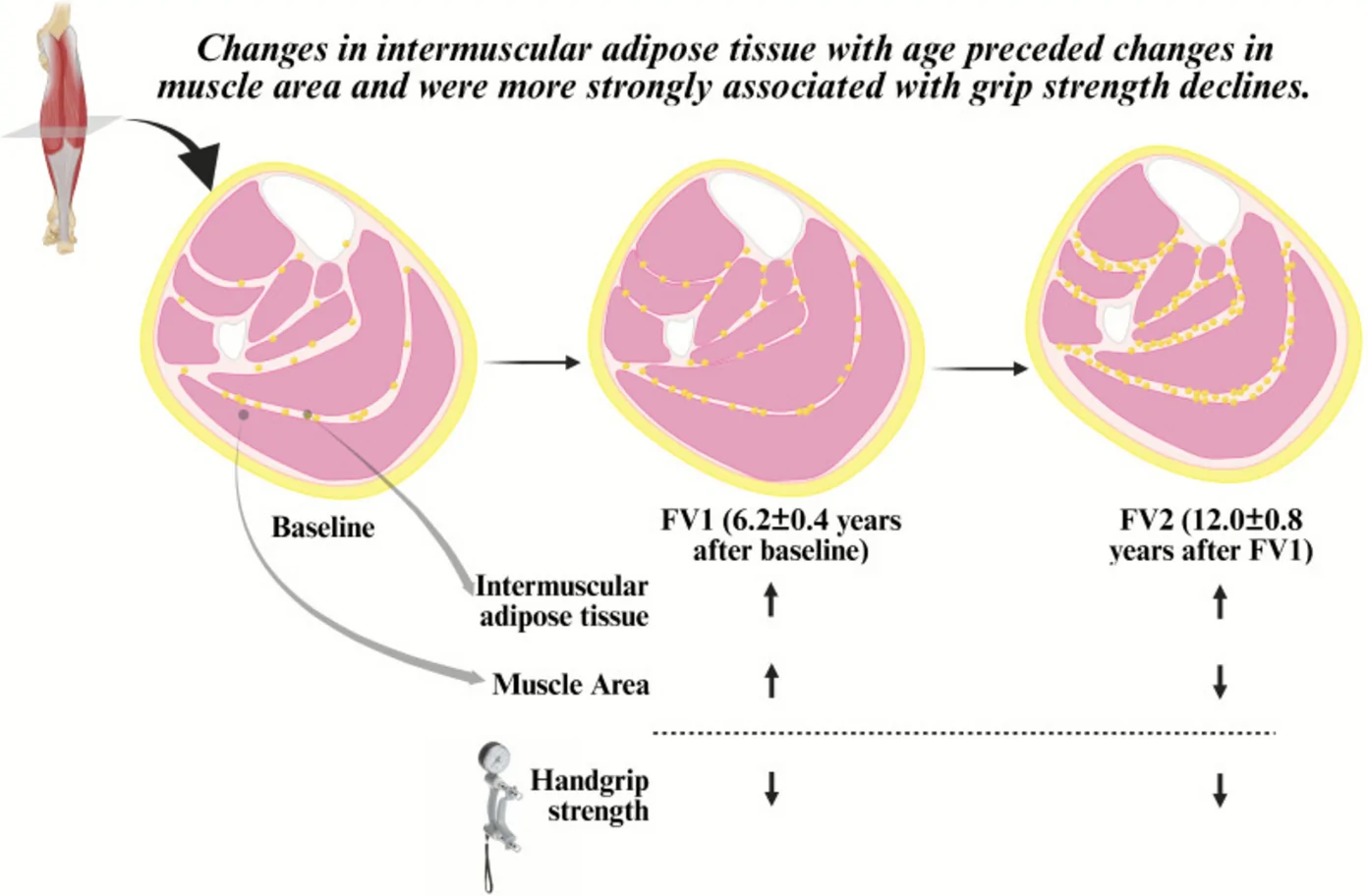

**Supplementary information:**

The online version contains supplementary material available at https://doi.org/10.1007/s11357-025-02023-8.

## Introduction

Skeletal muscle is central to mobility and metabolic function. Accordingly, loss of skeletal muscle mass and function with age has a tremendous impact on health, posing a global public health problem that is expected to increase substantially as the world’s population of older adults increases [1]. Muscle mass peaks between 30 and 40 years of age, then declines thereafter with dramatic reductions occurring after age 60 years [2], and declines are typically observed earlier in the lower than upper body [3]. However, skeletal muscle strength declines outpace muscle mass loss after age 50–60 years (2–4%/year vs. ~ 1%/year) [4, 5], contributing to the risk of several major age-related chronic conditions. Grip strength in particular is considered a “marker of aging,” as it can be indicative of current health status and a predictor of future events including death [6]. Furthermore, changes to skeletal muscle tissue are accompanied by the accumulation of ectopic adipose tissue within skeletal muscle (i.e., myosteatosis) [7].

Myosteatosis is an umbrella term describing the deposition of adipose tissue into various regions of skeletal muscle (intermuscular, intramuscular, and intramyocellular adipose tissue depots) [8]. We and others have found that persons of African ancestry have greater myosteatosis compared to White men at all levels of total adiposity [9, 10], and that increased myosteatosis is associated with several aging-related diseases and conditions, including muscle strength decline [11, 12], metabolic disorders [13], and mortality [14]. Thus, myosteatosis is an adiposity depot of broad importance for metabolic and musculoskeletal health, as well as successful aging and longevity. Despite this significance, there have been only a few longitudinal studies on myosteatosis, all showing that myosteatosis increases with age, independent of weight change [9, 15], and is associated with progressive muscle weakness [9], slowed gait speed [16], and incident mobility limitations [17]. However, all previous studies are limited to adults aged ≥ 65 years, have relatively short follow-up periods (2.5–5 years), and collected data only twice. The present analysis therefore characterized the natural history of and association with aging-related changes in muscle strength, size, and adiposity in community-dwelling African ancestry men aged ≥ 40 years over three follow-up visits spanning nearly two decades. We hypothesized that increases in intermuscular adipose tissue (IMAT) precede the loss of muscle area and muscle strength, and that a greater increase in IMAT is associated with accelerated loss of grip strength, independent of muscle area loss.

## Methods

### Study sample and design

The Tobago Health Study is a population-based, prospective cohort of predominantly African ancestry men aged 40 years and older in the Caribbean Island of Tobago, Republic of Trinidad and Tobago [18]. Briefly, men were initially recruited from 1997 to 2003 for prostate cancer screening, with follow-up assessments of body composition and related risk factors. To be eligible to enroll in the study, men had to be ambulatory, noninstitutionalized, and free from terminal illness. Between 2004 and 2007, men were invited to return for a whole body and skeletal muscle composition assessment where peripheral quantitative computed tomography (pQCT) was measured for the first time in the study (present analysis baseline visit; *n* = 2,152) [15]. Between 2010 and 2014, men with available pQCT scans from the previous visit were invited to return for an additional pQCT scan (present analysis follow-up visit 1 [FV1]; *n* = 1,515, 80% of surviving baseline men). Again in 2023 and 2024 (present analysis follow-up visit 2 [FV2]), a specific subset of men aged 65 years and older with calf pQCT and all cardiovascular disease measures collected previously were invited to return for an ancillary study that aimed to evaluate cognitive functioning in 430 men when additional pQCT scans were performed. A total of 339 men with grip strength measures and pQCT scans at the tibia at FV2 and at least one other tibia pQCT scan at baseline or FV1 were included in this analysis. The men included in this analysis tended to be slightly younger and were less likely to have hypertension, diabetes, or cancer at baseline than the men who were not included (sTable 1). The study was approved by the University of Pittsburgh and the Tobago Division of Health, Wellness and Social Protection Institutional Review Boards. All participants provided written and informed consent at each study visit.

### Peripheral quantitative computed tomography (pQCT) measured skeletal muscle composition

As described previously [15, 19], calf IMAT (cm^2^), cross-sectional muscle area (cm^2^), and subcutaneous adipose tissue (SAT; cm^2^) were obtained from pQCT scans at the tibia (Stratec XCT-2000 scanner; Orthometrix, Inc., White Plains, NY). Scans were obtained at 66% of the tibial length, proximal to the terminal end of the tibia using standard procedures for participant positioning and scan analysis [20]. The 66% of the tibial length site was selected because it has the largest circumference and the lowest variability in composition between individuals [21]. Tissues were distinguished according to density thresholds using the manufacturer’s suggested analysis parameters [20]. All image analyses and quality control at all three study visits were completed by a single investigator (C.L.G.) using Stratec analysis software version 6.20C (Orthometrix Inc.).

### Grip strength

Grip strength was measured by a Jamar handheld dynamometer (Sammons Preston Rolyan, Bolingbrook, IL, USA) using identical protocols at all three study visits. Participants were instructed to stand with the elbow of the hand being tested tucked to their side and their forearm extended at a 90-degree angle. Once in position, participants were asked to squeeze the dynamometer as hard as they could for a total of two times on each hand. The maximum measure of both hands was taken at each visit.

### Potential confounders

Lifestyle behaviors, demographics, and medical conditions were collected during an interviewer-administered questionnaire at baseline. Smoking was classified as never (smoked < 100 cigarettes or five packs in a lifetime and not a current smoker), former (ever smoked > 100 cigarettes or five packs, but not a current smoker), or current. Regular alcohol use was defined as consuming more than 4 units of any type of alcohol per week. Physical activity level was determined by self-reported duration of walking for leisure or exercise per week, as walking is the predominant form of physical activity on the island of Tobago. Hypertension (yes/no) was defined as having a systolic blood pressure ≥ 130 mmHg, a diastolic blood pressure ≥ 80 mmHg, or use of anti-hypertensive medication. Diabetes (yes/no) was defined as self-reported treatment of diabetes or fasting glucose ≥ 126 mmol/L. Medication for lipids was also recorded. Cardiovascular disease (yes/no) was defined as self-reported physician diagnosis of any one of the following: stroke, heart attack, angina, intermittent claudication, or rheumatic/valvular heart disease. Cancer history (yes/no) was defined as ever receiving a physician diagnosis of cancer, a malignant growth, or malignant tumor. Height (cm) was measured using a wall-mounted stadiometer. Body weight (kg) was measured at all three visits without shoes on a balance beam scale. Body mass index (BMI) was calculated as body weight divided by height squared (kg/m^2^).

### Statistical approach

Baseline demographic and health characteristics are presented as mean and standard deviation or n (%). Grip strength, IMAT, muscle area, and SAT at each follow-up visit were presented as mean and standard deviation or median (quartile 1, quartile 3), depending on normality, and absolute and percent change between visits was calculated. Change per year in grip strength was calculated since it changed linearly over time. Unadjusted and age-adjusted differences across visits were determined by repeated measures analysis of variance (PROC MIXED procedure in SAS) for grip strength, muscle area, and SAT, and by univariate generalized estimating equations (GEE; PROC GENMOD procedure in SAS) for IMAT since it was not normally distributed. Post-hoc tests were used to determine pairwise differences between visits if indicated.

A GEE was used to determine the association between change from baseline in IMAT and muscle area separately with grip strength (PROC GENMOD procedure in SAS). An unstructured correlation matrix was used to account for the within-subject variation. Grip strength declined linearly with time, so time was modeled continuously as years of follow-up from baseline. Model 1 adjusted for baseline IMAT or muscle area, baseline age (years), baseline height (cm), time-varying weight (kg), and follow-up time. Model 2 additionally adjusted for the following baseline characteristics and lifestyle behaviors: smoking status, regular alcohol use, cardiovascular disease, hypertension status, cancer history, diabetes status, and number of hours/week walking. In the model with IMAT as the exposure, time-varying muscle area was entered to determine if the association was independent of muscle size. A sensitivity analysis was run excluding participants with ≥ 3 standard deviations outside the mean across all three visits for IMAT (12.97 cm^2^; *n* = 11).

The difference in baseline characteristics, grip strength, IMAT, muscle area, and SAT was also evaluated by age group (< 50 years vs. ≥ 50 years). Statistical significance in baseline characteristics by age group was determined using a two-sample t-test for continuous variables or a chi-square test for categorical variables. Differences across time by age group were determined using an interaction term between age group and time in a linear mixed effects model (PROC MIXED procedure in SAS) for grip strength, muscle area, and SAT, and using GEE for IMAT. To determine if associations between change in IMAT and muscle area with grip strength varied by age group, an interaction term between age group and change in IMAT or area was included in the model. All analyses were conducted in SAS Version 9.4M7 (SAS Institute, Cary, NC, USA) and all figures were created in R Version 4.3.2 (R Core Team, 2023).

## Results

A total of 339 men were included in this analysis. Average follow-up time from baseline to FV1 and from FV1 to FV2 was 6.2 ± 0.4 and 12.0 ± 0.8 years, respectively, for a total average follow-up of 18.2 ± 0.9 years. At baseline, men were in their early 50 s (51.2 ± 5.5 years), overweight (average BMI: 27.5 ± 4.3 kg/m^2^), mostly never smokers (69.6%), and few were on lipid-lowering medication (1.8%; Table 1). The prevalence of cardiovascular disease (3.3%) and cancer (2.4%) was low, but there was a high prevalence of hypertension (63.3%) and type 2 diabetes (9.8%). Men walked an average of about 2.5 h/week, and few consumed four or more alcoholic drinks/week (11.2%). Participant health characteristics were similar by age group (< 50 vs. ≥ 50 and older), except that those aged < 50 years were more likely to be never smokers (*p* = 0.04; sTable 2).

**Table 1:** Baseline health characteristics of the Tobago Health Study men with pQCT at three time points

Participant characteristic	mean ± SD or *n* (%)
Age, years	51.2 ± 5.5
Standing height, cm	176.2 ± 6.7
Weight, kg	85.5 ± 14.1
BMI, kg/m^2^	27.5 ± 4.3
Hypertension	214 (63.3)
Type 2 diabetes	33 (9.8)
Taking a lipid lowering medication	4 (1.2)
Cardiovascular disease	11 (3.3)
Cancer history	8 (2.4)
Smoking status	
Never	236 (69.6)
Past	65 (19.2)
Current	38 (11.2)
Regular alcohol use	38 (11.2)
Walking hours/week	2.6 ± 4.7

*pQCT* peripheral quantitative computed tomography, *SD *standard deviation, *BMI* body mass index

Cardiovascular disease was defined as self-reported physician-diagnosis of any one of the following: stroke, heart attack, angina, intermittent claudication, or rheumatic/valvular hear disease. Cancer history was defined as a self-reported physician diagnosis of cancer, a malignant growth, or malignant tumor. Regular alcohol use is defined as ≥ 4 drinks/week

**Fig. 1:**
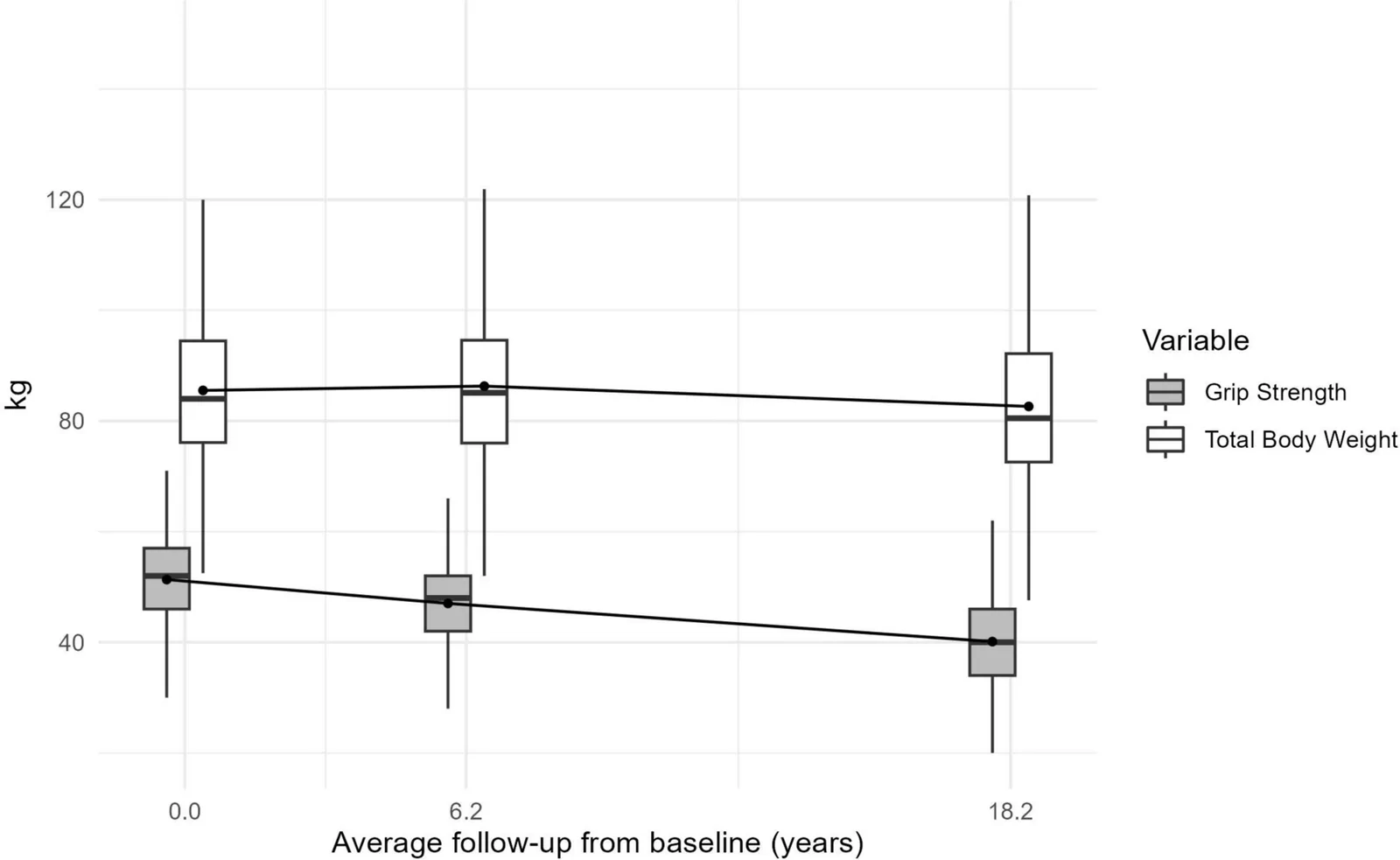
Grip strength and body weight (kg) over three time points in Tobago Health Study men

**Fig. 2:**
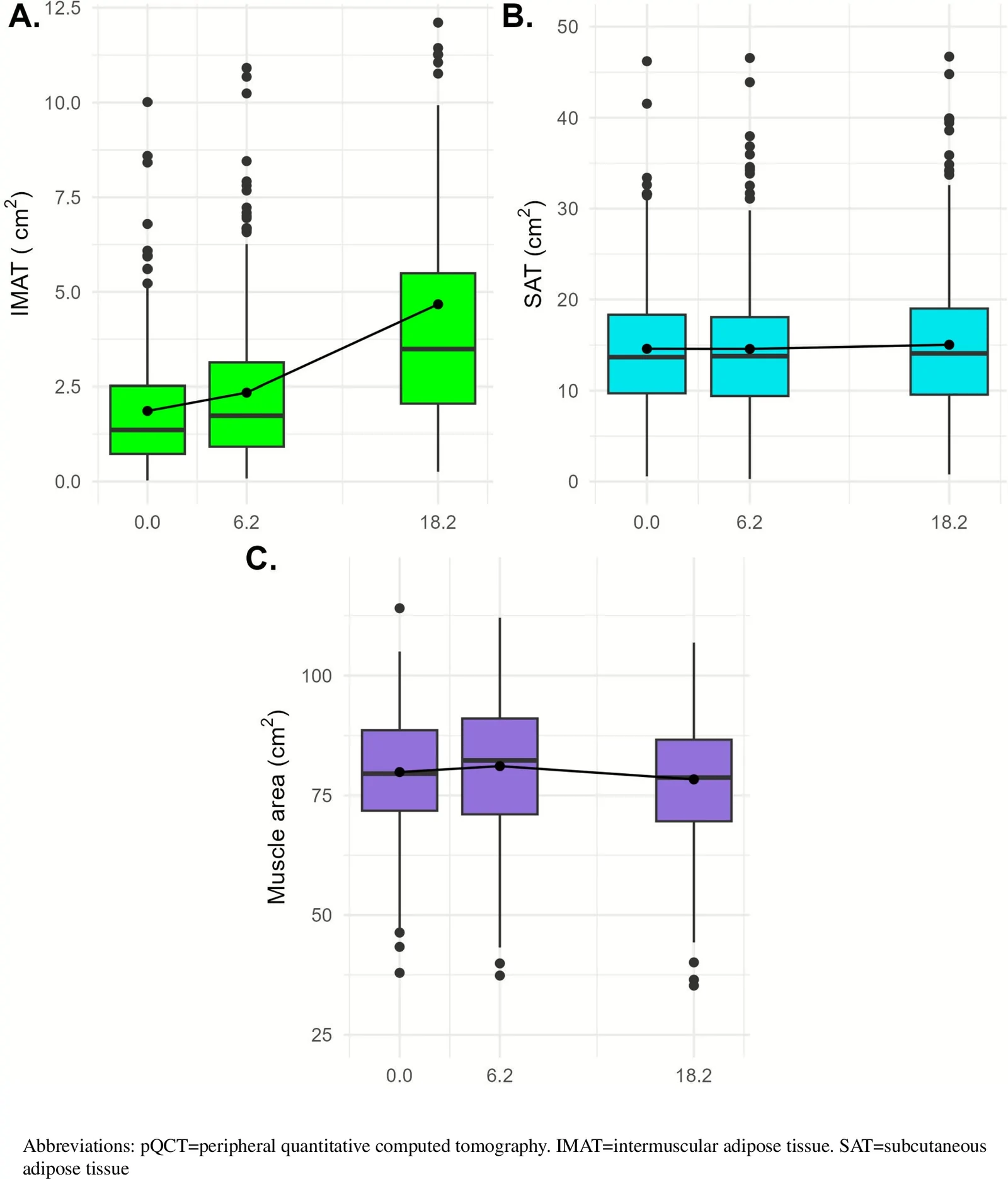
pQCT-measured calf IMAT, muscle area, and SAT (cm^2^) over three time points in Tobago Health Study men. Abbreviations: pQCT=peripheral quantitative computed tomography. IMAT=intermuscular adipose tissue. SAT=subcutaneous adipose tissue

Overall, grip strength and muscle area significantly decreased over time, and IMAT significantly increased over time (both *p* < 0.001; Table 2). Grip strength declined by 1.3 ± 1.9% per year from baseline to FV1, and by 1.2 ± 1.1% per year from FV1 to FV2 (Fig. 1). Conversely, IMAT increased from baseline to FV1 by a median of 0.25 (quartile 1, quartile 3: −0.38, 1.15) cm^2^, and from FV1 to FV2 by a median of 1.47 (0.57, 2.94) cm^2^ (Fig. 2). Muscle area increased from baseline to FV1 (0.94 ± 6.63 cm^2^), then decreased from FV1 to FV2 (−4.02 ± 8.30 cm^2^; Fig. 2). No significant changes across time were observed for SAT (*p* = 0.14).

**Table 2:** Numerical summary of grip strength, and calf muscle area and adipose tissue at each study visit

Mean ± SD or median (Q1, Q3)	Baseline	FV1	FV2	*p*-value
Grip strength, kg	51.33 ± 8.79	47.02 ± 8.67^a^	40.12 ± 8.57^a,b^	< 0.001
Muscle composition				
IMAT, cm^2^	1.36 (0.73, 2.53)	1.74 (0.92, 3.14)^c^	3.49 (2.06, 5.49)^c,d^	< 0.001
Subcutaneous fat, cm^2^	14.58 ± 6.86	14.56 ± 7.18	15.02 ± 7.94	0.14
Muscle area, cm^2^	79.88 ± 12.33	81.12 ± 13.88^a^	78.35 ± 12.72^a,b^	< 0.001

*SD* standard deviation, *Q* quartile, *FV* follow-up visit, *IMAT* intermuscular adipose tissue

Unadjusted and age-adjusted p-value across visits determined by repeated measures analysis of variance for grip strength, subcutaneous fat and muscle area, and by univariate generalized estimating equation for IMAT. Unadjusted and age-adjusted p-values were the same for all measures

^a^*p* < 0.05 from baseline using post-hoc test with Tukey-Kramer adjustment

^b^*p* < 0.05 from FV1 using post-hoc test with Tukey-Kramer adjustment

^c^*p* < 0.05 from baseline using post-hoc test of least squares means

^d^*p* < 0.05 from FV1 using post-hoc test of least squares means

There was a significant interaction between visit and age group (< 50 vs. ≥ 50 years) for mean grip strength (*p* = 0.004), but not for IMAT (*p* = 0.76), SAT (*p* = 0.47), or muscle area (*p* = 0.07; sTable 3). Changes in grip strength, IMAT, and muscle area by age group followed a similar general pattern to one another (sFig. 1–3). However, the absolute change from FV1 to FV2 for those aged ≥ 50 years compared to those aged < 50 years was greater for grip strength (–7.6 ± 6.0 vs. –5.9 ± 61 kg; *p* = 0.01) and muscle area (–4.97 ± 8.25 cm^2^ vs. –2.85 ± 8.25 cm^2^; *p* = 0.03). For IMAT, the median change from FV1 to FV2 was less for those aged ≥ 50 years compared to those aged < 50 years (0.26 [–0.33, 1.24] vs. 1.32 [0.52, 2.58] cm^2^; *p* = 0.03).

Results from the GEE showed that a 1-cm^2^ increase in IMAT from baseline to FV1 or FV2 was associated with a 0.21 kg (95% CI: −0.34, −0.08) decrease in grip strength in model 2 (Table 3). Results remained unchanged when muscle area was added to the model (*β* = −0.18, 95% CI: −0.31, −0.06). Excluding anyone with ≥ 3 standard deviations outside of the mean for IMAT (*n* = 11) did not meaningfully change results (sTable 4); the unadjusted model was not significant, but once time-varying weight was included, the model was significant, and the magnitude of the association was stronger than when outliers were included.

**Table 3:** Association between a 1-cm^2^ change in IMAT or muscle area with time-varying grip strength

	Change in IMAT		Change in muscle area	
	*β* (95% CI)	*p*-value	*β* (95% CI)	*p*-value
Unadjusted	−0.14 (−0.28, −0.0003)	0.0495	0.07 (0.007, 0.13)	0.03
Model 1^a^	−0.20 (−0.33, −0.08)	0.002	0.04 (−0.03, 0.10)	0.26
Model 2^b^	−0.21 (−0.34, −0.08)	0.002	0.03 (−0.03, 0.10)	0.35
Model 2 + muscle area	−0.18 (−0.31, −0.06)	0.005	–	–

*IMAT* intermuscular adipose tissue, *CI* confidence interval

^a^Model 1: baseline IMAT or muscle area, baseline age, baseline height, time-varying body weight, and follow-up time

^b^Model 2: model 1 + baseline lifestyle behaviors and health characteristics: smoking (never vs. past or current), regular alcoholic use (≥ 4 drinks/week; yes/no), cardiovascular disease (history of stroke, heart attack, angina, intermittent claudication, rheumatic/valvular heart disease; yes/no), hypertension (yes/no), cancer (self-reported history of cancer, a malignant growth, or malignant tumor; yes/no), diabetes (self-reported treatment of diabetes or fasting glucose ≥ 126 mmol/L; yes/no), walking hours/week

A 1-cm^2^ decrease in muscle area was significantly associated with decreased grip strength in the unadjusted model only, but results were completely attenuated and not significant when covariates were added for models 1 and 2. There was no significant interaction between age group and change in IMAT or change in muscle area (all *p* > 0.05; data not shown).

## Discussion

In this cohort of middle-aged to older African Caribbean men, we found a significant age-related increase in IMAT over an average of almost two decades of follow-up. Over time, IMAT consistently increased, while muscle strength consistently and steadily decreased. However, SAT remained relatively unchanged over time, while muscle area increased then decreased during later ages. Greater increases in IMAT were significantly associated with decreased grip strength, independent of baseline IMAT, age, weight, time, and muscle area. Moreover, despite the smaller magnitude of change in IMAT over time compared to muscle area, the association between IMAT and grip strength was stronger than that of muscle area and grip strength. This suggests a stronger influence of skeletal muscle adiposity vs. size on muscle strength. These results provide novel evidence of the natural history and pattern of muscle size and adiposity changes over three time points to demonstrate that age-related changes to skeletal muscle adiposity, specifically IMAT, impact strength declines.

The observed increase in IMAT with age is supported by earlier work. Cross-sectionally, a significant correlation was observed between thigh IMAT and age (age range 18–87 years; *r* = 0.47; *p* < 0.001) in a study comparing older vs. younger adults [22]. Significant trends by 5-year [23] and 10-year [24] age groups were also observed in studies of older adults. The Health, Aging and Body Composition (Health ABC) study previously examined 5-year changes in myosteatosis in older adults and reported that mid-thigh IMAT increased with age among African-American and White men and women separately [9]. However, participants in this study were all recruited to be 70–79 years old and were healthy and high-functioning. A prior analysis in the Tobago Health Study reported the annualized rate of increase in calf IMAT (11.3%/year) among African ancestry men aged 65 years and older to be similar to the annualized rate of increase in thigh IMAT reported by the abovementioned Health ABC study among men (9.7%/year) [15]. Our findings are in line with these prior results and add to our knowledge of the natural history of IMAT to show that increases in IMAT persist over nearly two decades, much longer than previously shown.

Prior longitudinal studies have also shown that increases in IMAT, particularly at the thigh and calf, over time are associated with poor muscle function. Over four years of follow-up in Health ABC, changes in IMAT were associated with decreased gait speed in Black and White men and women combined [16]. A separate Health ABC analysis found that 5-year increases in thigh IMAT coincided with progressive muscle weakness (measured by isokinetic leg muscle torque) in weight-stable men [9]. In the Tobago Health Study, gaining or maintaining IMAT over 7 years was associated with a higher likelihood of developing incident mobility limitations [17]. However, these studies are limited to adults aged 60 years or older and only included two time points, limiting our understanding of the natural history and trajectories of muscle adiposity and its association with grip strength during aging. Results from the present study provide novel pieces to the story by showing that muscle adiposity increases start in middle age and continue to increase in older adulthood, so this association with muscle function begins earlier than prior longitudinal studies showed.

While IMAT increased starting in middle age, muscle area did not begin to decline until men were slightly older. Grip strength declined throughout the follow-up period, despite the initial marginal increase in muscle area, suggesting there is a disconnect between muscle mass and muscle strength. These results support the seminal findings from the Health ABC cohort, which showed a 1% per year decrease in muscle mass but a 4% per year decrease in muscle strength after 3 years of follow-up [5]. Our findings suggest the increase in IMAT may be partially responsible for this disconnect. Given that IMAT starts to change before muscle area, this may also explain why we observed a stronger association between IMAT and grip strength compared to the association between muscle area and grip strength, as indicated by the larger beta coefficient.

The age range of our sample at study entry was 41–73 years, but we surprisingly did not observe any significant interactions with age group in the association between IMAT and muscle area with grip strength. Those aged 50 years and older had a slightly greater decrease in grip strength over time compared to those younger than 50 years, so we anticipated a slight difference in the observed association by age group. However, we may have been underpowered to detect a significant difference by age group. Additionally, while we had an age range spanning over three decades, we did not have a large enough sample size to evaluate older vs. younger adults (e.g., < 65 years vs. ≥ 65 years). Instead, we had power to test middle age vs. older middle age to older adults at baseline, so results may have been different if we could compare older vs. younger adults.

The exact biological mechanisms behind increased IMAT with age are unknown, but there are several biological pathways that contribute to the observed increase in IMAT. With age, leptin signaling (a hormone important for regulating eating behaviors and energy balance) becomes increasingly defective, which can decrease fatty acid metabolism and increase muscle adiposity [25]. There is also a sex steroid deficiency with age, particularly testosterone [26] and glucocorticoid treatment [27] that may cause fatty infiltration into the muscle. Several lifestyle factors tend to change with age, including diet and exercise. The changes in physical activity and timing of nutrient intake can impact circadian rhythms, influencing clock genes in muscles, which may subsequently increase myosteatosis [8].

There are also several proposed biological pathways through which this age-related increase in IMAT impacts muscle function. It is believed that IMAT impacts muscle function directly (vs. being a physiological consequence) through one of several different potential pathways [28]. The close proximity of fat to muscle fibers may impair the local muscle environment [29] through increases in local or systemic proinflammatory cytokines [30] or impaired myokine secretion [31]. Another mechanism may be through impaired mitochondrial function that occurs in a state of lipotoxicity through reduced expression of PGC-1α, which is involved in mitochondrial biogenesis, or through oxidative damage that harms mitochondrial DNA and RNA [32]. IMAT produces a greater inflammatory response vs. SAT, which may contribute to lower strength [12]. Recent evidence shows that IMAT has a robust plasma proteomic profile that is associated with lower central processing speed [33], so this proteomic profile may influence other key areas of the muscle and central nervous system that are important for strength. Adipose tissue in the muscle can alter the mechanics of the muscle by impairing muscle fiber orientation, fascicle length, and possibly lead to a lower pennation angle [34]. IMAT is also strongly associated with insulin resistance and type 2 diabetes, suggesting that it has adverse metabolic consequences that may also translate to poor muscle strength [8, 13]. While evidence from our study shows the natural history of myosteatosis with age, there is a lack of knowledge on the corresponding biological changes in middle age to older adulthood over extended periods of time, limiting our understanding of possible biological pathways responsible for these changes over extended periods of time.

Our study is strengthened by evaluating changes in muscle composition and strength over three time points and including both middle-aged and older adults, allowing us to observe the natural history of changes over nearly two decades. This study also followed standard protocols for evaluation of muscle tissue segmentation, and measures were all evaluated by the same person across all three visits. We only measured IMAT at one anatomical location, so results may not be generalizable to IMAT at other anatomical locations. However, single CT images of the skeletal muscle are commonly used as a proxy for whole body skeletal muscle [35], and myosteatosis at the thigh and calf are strongly correlated [36], suggesting results may be applicable to other lower body myosteatosis measures. Myosteatosis at the calf also is more strongly associated with grip strength compared to other anatomical sites such as the abdomen [11], and thus, the anatomical location we analyzed is an important determinant of grip strength.

We were limited by sample size; our sample size was not large enough to evaluate an interaction between older adults vs. younger adults (< 65 vs. ≥ 65 years). This sample is also subject to survivor bias, since only a subset was brought back for an ancillary study with an objective of studying cognitive function. Additionally, grip strength was used as a measure of total body muscle strength. While there is a significant correlation (*r* = 0.69, *p* < 0.001) between hand grip strength measured by a dynamometer and global muscle strength measured by an isokinetic dynamometer at the trunk, hip, knee, and ankle, this interpretation of grip strength as a surrogate for total body strength should be used with caution since grip strength may not track with strength at other skeletal muscle groups [37]. Calf myosteatosis is significantly associated with other functional measures like chair rise time and gait speed [11], as well as incident mobility limitations [17]. This suggests that results could be generalizable to other functional measures, but future longitudinal work would need to validate these results longitudinally, as data on other measures were not available in this study. We only had self-reported walking hours/week for physical activity, so other types of activity, objective measures of activity, or activity intensity may have different effects than time spent walking. Further, diet, a potentially important lifestyle determinant of adiposity, was not directly assessed in our study. Finally, our cohort also included only middle-aged and older African ancestry men, and thus, findings may not apply to women and other populations. Future studies should include women and populations living outside of the Caribbean region.

In conclusion, lower leg skeletal muscle tissue distribution changes over time, starting in middle age with increases in IMAT and followed by decreases in muscle area in older adulthood. Associations between increases in IMAT and decreases in grip strength are stronger than those of decreases in muscle area and decreases in grip strength, likely because of the earlier changes in IMAT compared to muscle area. Finally, changes in IMAT over almost two decades are associated with progressive muscle weakness, independent of muscle size, body weight, age, and time. Results from this study support the notion that IMAT may be an independent marker of aging that may be responsible for the disconnect between age-related declines in muscle mass and muscle strength.

## Supplementary information

Below is the link to the electronic supplementary material.

ESM 1 (DOCX 139 KB)

## Data Availability

De-identified data may be requested from the Tobago Health study with an approved data use agreement through the University of Pittsburgh. The statistical code used to generate the results is available upon request from the corresponding author.
