# Supplementary material for: Age-related change in skeletal muscle strength, size, and adiposity over 18 years in Caribbean men
Source: GeroScience. 2025 Dec 10;48(5):7039–49. doi: 10.1007/s11357-025-02023-8 (PMC13601445; doi:10.1007/s11357-025-02023-8)
Supplement: Supplementary file 1 [file 11357_2025_2023_MOESM1_ESM.docx]

**sTable 1** Baseline health characteristics of men enrolled in the Tobago Health Study that did not return for pQCT scans.

| **Participant characteristic at baseline (n=2,143)** | **mean±SD or or n(%)** | **p-value compared to those included** |
| --- | --- | --- |
| Age, years | 60.1±10.5 | <0.001 |
| Standing height, cm | 174.8±6.9 | <0.001 |
| Weight, kg | 84.4±16.9 | 0.18 |
| BMI, kg/m^2^ | 27.6±5.0 | 0.87 |
| Hypertension | 1525 (71.5) | 0.002 |
| Type 2 diabetes | 447 (22.4) | <0.001 |
| Cardiovascular disease | 107 (5.1) | 0.14 |
| Cancer history | 190 (9.1) | <0.001 |
| Smoking Status |  |  |
| Never | 1389 (66.2) | 0.20 |
| Past | 494 (23.6) |  |
| Current | 214 (10.2) |  |
| 4+ drinks/week | 229 (10.9) | 0.86 |
| Walking hours/week | 3.1±6.1 | 0.20 |

Abbreviations: pQCT=peripheral quantitative computed tomography. SD=standard deviation. BMI=body mass index.

*Note.* Cardiovascular disease was defined as self-reported physician-diagnosis of any one of the following: stroke, heart attack, angina, intermittent claudication, or rheumatic/valvular hear disease. Cancer history was defined as a self-reported physician diagnosis of cancer, a malignant growth, or malignant tumor. Statistical difference compared those included in the analysis to those excluded (e.g., without follow-up pQCT) using independent sample t-test for continuous variables or chi-square test for categorical variables.

**sTable 2** Baseline health characteristics by age group (<50 vs. ≥50 years) of participants men in the Tobago Health Study with pQCT scans.

| mean±SD or n(%) | **<50 years (n=150)** | **≥50 years (n=189)** | **p-value** |
| --- | --- | --- | --- |
| Age, years | 46.4±1.7 | 55.0±4.5 | <0.001 |
| Standing height, cm | 176.0±6.2 | 176.3±7.1 | 0.66 |
| Weight, kg | 85.9±13.6 | 85.2±14.5 | 0.64 |
| BMI, kg/m^2^ | 27.7±4.0 | 27.4±4.6 | 0.59 |
| Hypertension | 88 (58.7) | 126 (67.0) | 0.11 |
| Type 2 diabetes | 16 (10.7) | 17 (9.0) | 0.62 |
| Cardiovascular disease | 2 (1.3) | 9 (4.8) | 0.12 |
| Cancer | 2 (1.3) | 6 (3.2) | 0.31 |
| Smoking Status |  |  |  |
| Never | 110 (73.3) | 126 (66.7) | 0.04 |
| Past | 20 (13.3) | 45 (23.8) |  |
| Current | 20 (13.3) | 18 (9.52) |  |
| 4+ drinks/week | 17 (11.3) | 21 (11.2) | 0.96 |
| Walking hours/week | 1.5 (0.0, 4.0) | 0.67 (0.0, 3.50) | 0.19 |

Abbreviations: pQCT=peripheral quantitative computed tomography. SD=standard deviation. BMI=body mass index

*Note.* Cardiovascular disease was defined as self-reported physician-diagnosis of any one of the following: stroke, heart attack, angina, intermittent claudication, or rheumatic/valvular hear disease. Cancer was defined as a self-reported physician diagnosis of cancer, a malignant growth, or malignant tumor. P-value assessed by independent sample t-test for continuous variables, or chi-square test for categorical variables.

**sTable 3** Numerical summary of muscle strength, size, and adipose tissue at each study visit by age group (<50 vs. ≥50 years).

| mean±SD or median [Q1, Q3] | **Age group** | **Baseline** | **FV1** | **FV2** | ***P-value** |
| --- | --- | --- | --- | --- | --- |
| Grip strength, kg | <50 | 53.52±8.79 | 49.58±9.34 | 43.87±8.66 | 0.004 |
|  | ≥50 | 49.59±8.42 | 44.97±7.51 | 37.19±7.28 |  |
| IMAT, cm2 | <50 | 1.38 (0.75, 2.52) | 1.62 (0.90, 2.84) | 3.78 (1.96, 5.81) | 0.76 |
|  | ≥50 | 1.29 (0.72, 2.53) | 1.84 (0.92, 3.24) | 3.37 (2.13, 5.40) |  |
| Subcutaneous fat, cm2 | <50 | 15.13±6.84 | 15.15±7.40 | 15.28±7.78 | 0.47 |
|  | ≥50 | 14.15±6.85 | 14.10±6.98 | 14.82±8.08 |  |
| Muscle area, cm2 | <50 | 81.62±12.56 | 83.16±14.43 | 81.08±12.37 | 0.07 |
|  | ≥50 | 78.48±11.99 | 79.49±13.23 | 76.15±12.61 |  |

Abbreviations: SD=standard deviation. Q=quartile. FV=follow-up visit. IMAT=intermuscular adipose tissue.

***P-value testing for an interaction between time and age group using PROC MIXED (grip strength, subcutaneous fat, muscle area) or PROC GENMOD (IMAT).

**sTable 4** Association between a 1-cm^2^ change from baseline in IMAT with time-varying grip strength, outliers excluded (IMAT≥12.97cm^2^).

|  | Change in IMAT | |
| --- | --- | --- |
|  | β (95% CI) | p-value |
| Unadjusted | -0.18 (-0.41, 0.06) | 0.14 |
| Model 1^a^ | -0.31 (-0.55, -0.07) | 0.01 |
| Model 2^b^ | -0.31 (-0.55, -0.08) | 0.01 |
| Model 2 + muscle area | -0.30 (-0.53, -0.07) | 0.01 |

Abbreviations: IMAT=intermuscular adipose tissue. CI=confidence interval.

^a^Model 1: baseline IMAT or muscle area, baseline age, baseline height, time-varying body weight, and follow-up time.

^b^Model 2: Model 1 + baseline lifestyle behaviors and health characteristics: smoking (never vs. past or current), ≥4 alcoholic drinks/week (yes/no), cardiovascular disease (history of stroke, heart attack, angina, intermittent claudication, rheumatic/valvular heart disease; yes/no), hypertension (yes/no), cancer (self-reported history of cancer, a malignant growth, or malignant tumor; yes/no), diabetes (self-reported treatment of diabetes or fasting glucose ≥126 mmol/L; yes/no), walking hours/week.

**sFig. 1** Average grip strength (kg) over three time points by age group (<50 vs. ≥50 years) in Tobago Health Study men.

Abbreviations: Abs=absolute. Abs Ann=absolute annual. Δ=change.

**sFig. 2** Average IMAT over three time points by age group (<50 vs. ≥50 years) in Tobago Health Study men.

Abbreviations: IMAT=intermuscular adipose tissue. Abs=absolute; Δ=change.

**sFig. 3** Average muscle area over three time points by age group (<50 vs. ≥50 years) in Tobago Health Study men.

Abbreviations: Abs=absolute. Δ=change.
